# Characterization and immune regulation role of an immobilization antigen from *Cryptocaryon irritans* on groupers

**DOI:** 10.1038/s41598-018-25710-3

**Published:** 2019-01-31

**Authors:** Ze-Quan Mo, Shun Xu, Donna M. Cassidy-Hanley, Yan-Wei Li, Daniel Kolbin, Jennifer M. Fricke, An-Xing Li, Theodore G. Clark, Xue-Ming Dan

**Affiliations:** 1 Joint Laboratory of Guangdong Province and Hong Kong Regions on Marine Bioresource Conservation and Exploitation, College of Marine Sciences, South China Agricultural University, Guangzhou, 510642 China; 2 School of Bioscience and Bioengineering, South China University of Technology, Guangzhou, 510006 PR China; 3 Department of Microbiology and Immunology, College of Veterinary Medicine, Cornell University, Ithaca, NY 14853 USA; 4 State Key Laboratory of Biocontrol/ Key Laboratory of Aquatic Product Safety (Sun Yat-Sen University), Ministry of Education, The School of Life Sciences, Sun Yat-sen University, Guangzhou, 510275 Guangdong Province PR China

## Abstract

Immobilization antigens (i-antigens) are surface membrane proteins that are widely recognized to be the ideal candidates as vaccines antigens for immunization against *Cryptocaryon irritans*. In this study, we cloned a putative i-antigen gene from *C. irritans*, which was expressed in all three stages of the *C. irritans* life-cycle, and localized primarily to the cell surface. The recombinant GDCI3 i-antigen was expressed and purified using the free-living ciliate, *Tetrahymena thermophila* as an expression system. The purified recombinant protein was recognized by rabbit anti-*C. irritans* antiserum and was capable of eliciting immobilizing antibodies in rabbits and fish suggesting that the antigen itself was correctly folded. Following immunization and parasite challenge, groupers vaccinated with, recombinant GDCI3 i-antigen had a 25% cumulative percent survival rate compared to 8.3% for controls. Both non-specific and parasite-specific IgMs were generated in fish following immunization, with the levels of both increasing following challenge. Parasite-specific IgM in mucus could only be elicited after challenge of the GDCI3 i-antigen vaccinated groupers. To our knowledge, this is the first report using the *Tetrahymena* expression system to generate *C. irritans* i-antigens and investigate their use for fish vaccination.

## Introduction

*Cryptocaryon irritans* is an obligate parasitic ciliate that infects numerous species of saltwater fish causing marine white spot disease^1^. To date there is no effective way to control cryptocaryonosis and destructive economic losses tied to the disease are not uncommon. Previous studies have shown that sublethal infections with *C. irritans* can elicit protective resistance^2–5^, and an array of host immune responses that include chemokine synthesis, activation of Toll-like receptor (TLR) signaling, mobilization of phagocytes, activation of nonspecific cytotoxic cells, and signaling through B- and T-cell receptors^6–11^. These findings suggest that vaccination may be an effective way to control *C. irritans* in an aquaculture setting. Our group successfully cultured the *C. irritans* and produced an inactivated whole cell (theront) vaccine that not only elicited specific antibodies, but provided protection in groupers against lethal parasite challenge^4,5^. Nevertheless, *C. irritans* is difficult to propagate outside the host^12,13^ and while it is possible to grow parasites in association with fish^14^, yields are limited, and mass culture of *C. irritans* for commercial vaccine development is costly and impractical.

Immobilization antigens (i-antigens) are surface membrane proteins originally identified in *Paramecium* and *Tetrahymena*, where they are differentially expressed in response to different environmental stimuli^15,16^. Structurally, i-antigens contain a glycosylphosphtidylinositol (GPI) modification at their C-termini, which anchors them to plasma and ciliary membranes^17,18^. I-antigens have been extensively studied in *Ichthyophthirius multifiliis*, a freshwater counterpart of *C. irritans*^19,20^. In this case i-antigens elicit strong antibody responses in infected fish and are considered likely vaccine candidates^20–22^. Antibodies from immune fish immobilize *Ichthyophthirius in vitro*, and juvenile channel catfish are completely protected against infection following passive immunization with i-antigen-specific monoclonal antibodies^22,23^. More recently, i-antigens have been identified in *C. irritans* as well^24–26^. Indeed, a DNA vaccine encoding one such antigen has recently been shown to protect fish against parasite challenge, and was strongly enhanced by the addition of a coding sequence for *C. irritans* HSP70^27,28^. Because regulatory hurdles for DNA vaccines remains relatively high in China and other countries, recombinant proteins offer a logical alternative. Nevertheless, i-antigens tend to be highly disulfide bonded, and the inability to produce these proteins in their native conformation in bacterial cells has been a major challenge^29^. To address this issue, we have begun to explore *Tetrahymena thermophila* as an alternate expression system for recombinant parasite antigens^30^. *Tetrahymena* grows to high density in inexpensive culture media and devotes a large part of its metabolism towards membrane protein production owing to its hundreds of cilia. Recently, i-antigens from *Ichthyophthirius* have been successfully expressed in *Tetrahymena* as correctly folded proteins that traffic to plasma and ciliary membranes^30^. Considering yield, biological activity and cost-effectiveness of i-antigen production in this system, *Tetrahymena* may offer a commercially viable platform for the manufacture of *C. irritans* vaccines.

In a previous study, analysis of mRNA transcripts from all three stages of *C. irritans*, including tomont, theront and trophont revealed nine predicted immobilization antigen ESTs^31^. Among these we selected the transcript with highest abundance levels in *C. irritans* as the candidate i-antigen (GDCI3) for expression in *Tetrahymena*. The selected cDNA was successfully expressed in the *Tetrahymena* expression system and its corresponding protein later purified. Recombinant GDCI3 i-antigen was recognized by rabbit anti-*C. irritans* antibody and was able to induce antibodies in rabbits and groupers that immobilized *C. irritans* theronts in culture. Immune protection and IgM antibody generated by vaccination with the GDCI3 i-antigen were further analyzed.

## Materials and Methods

### Ethics Statement

All animal protocols were reviewed and approved by the Animal Administration and Ethics Committee of College of Marine Sciences, South China Agricultural University. The study was performed in strict compliance with the recommendations set forth in the Animal Ethics Procedures and Guidelines of the People’s Republic of China. All efforts were made to minimize animal suffering and to reduce the numbers of animals used in the experiments.

### Cloning of GDCI3 I-antigen gene

Transcriptomic analysis of *C. irritans*, including RNAs from tomont, theront and trophont stages has previously been reported^31^. Annotation of the unigene set revealed transcripts for nine immobilization antigens including one for the GDCI3, which appeared to be the most highly expressed (S. Table 1). Analysis of ESTs suggested that the assembled GDCI3 transcript was less than full length and was missing sequence at its 5′-end. To determine that sequence, 5′-RACE,was conducted with the primers GDCI3 GSP1 F and GDCI3 GSP2 F (Table 1). Total RNA was extracted from theronts using TRIzol Reagent (Invitrogen), and cDNA was synthesized with the SMARTerTM RACE cDNA Amplification Kit (Clontech) following the manufacturers’ protocol. The RACE amplification protocol for both the primary and nested PCR was performed as follows: (98 °C for 10 s, 68 °C for 15 s, 72 °C for 2 min) × 35 cycles, 72 °C for 5 min for 1 cycle. Gene-specific primers GDCI3 F/R (Table 1) were designed to amplify the entire open reading frame (ORF) of the GDCI3 i-antigen gene. The amplification protocol was performed as follows: (98 °C for 10 s, 55 °C for 15 s, 72 °C for 2 min) × 35 cycles, 72 °C for 5 min for 1 cycle. All amplification products were purified and ligated to pEASY-Blunt Cloning Vector (TRANS, Beijing, China) for sequencing.

**Table 1:** Primers used in this study.

Primer	Sequence (5′ to 3′)
GDCI3 GSP1 F	AGTTTATCTTTACAATCACCAGTAGCAG
GDCI3 GSP2 F	TGTACCAACCCATGTAGCTTTTGTATTG
GDCI3 F	ATGTAAAAGATTTTAGCTATTTTATT
GDCI3 R	TCATTTGAATAAAAGAGCAAAG
GDCI3 RTF	CGATGATGAATGTGTAGTTGGAA
GDCI3 RTR	AGTATCTAAGGCTGTATCTCTATTGAA
EF-1 β RTF	GGAGATGATGATGATAATGATGA
EF-1 β RTR	CCAAACTAAACCTTCCAACT
UPM	Long: CTAATACGACTCACTATAGGGCAAGCAGTGGTATCAACGCAGAGT
	Short: CTAATACGACTCACTATAGGGC
NUP	AAGCAGTGGTATCAACGCAGAGT

### Sequence analysis of GDCI3 I-antigen

The ORF of GDCI3 i-antigen was searched by ORF finder (https://www.ncbi.nlm.nih.gov/orffinder/). The theoretical molecular weight was predicted using Compute pI/Mw tool (http://web.expasy.org/compute_pi/). Signal peptide was predicted using SignalP server (http://www.cbs.dtu.dk/services/SignalP). Transmembrane region was predicted using TMHMM Server (http://www.cbs.dtu.dk/services/TMHMM/). N-glycosylation sites and O-glycosylation sites were predicted with NetNGlyc 1.0 Server (http://www.cbs.dtu.dk/services/NetNGlyc) and NetOGlyc 4.0 Server (http://www.cbs.dtu.dk/services/NetOGlyc/), respectively. Glycosylphosphatidylinositol (GPI) anchored protein and ω-site were predicted with GPI Prediction Server (http://mendel.imp.ac.at/sat/gpi/gpi_server.html). The differences of codon usage preference between *C. irritans* and *Tetrahymena* were analyzed with GCUA tool (http://gcua.schoedl.de).

### Expression analysis of GDCI3 i-antigen gene

Total RNA isolation and subsequent cDNA synthesis were performed on samples of tomont, theront and trophont as described above. Expression levels of GDCI3 i-antigen transcripts at each stage were determined using the SYBR Green Realtime PCR Master Mix (Toyobo) according to manufacturer’s instructions. The GDCI3 RTF/R primers (Table 1) were used as gene-specific primers in real-time PCR, and elongation factor 1-beta (EF-1 β) primers were used as the reference gene. The cycling protocol was 94 °C for 2 min, and (94 °C for 15 s, 58 °C for 15 s, 72 °C for 20 s) × 40 cycles. Melting curve analysis was used for detecting the specificity of PCR products. PCR products were verified by sequencing. All samples were done in triplicate. The expression of the target gene were normalized to the reference gene calculating with the 2^−ΔΔCt^ method^32^. All data were analyzed using SPSS (version 16.0) software and expressed as Mean ± SE.

### Construction of transformation plasmids

**Figure 1:**
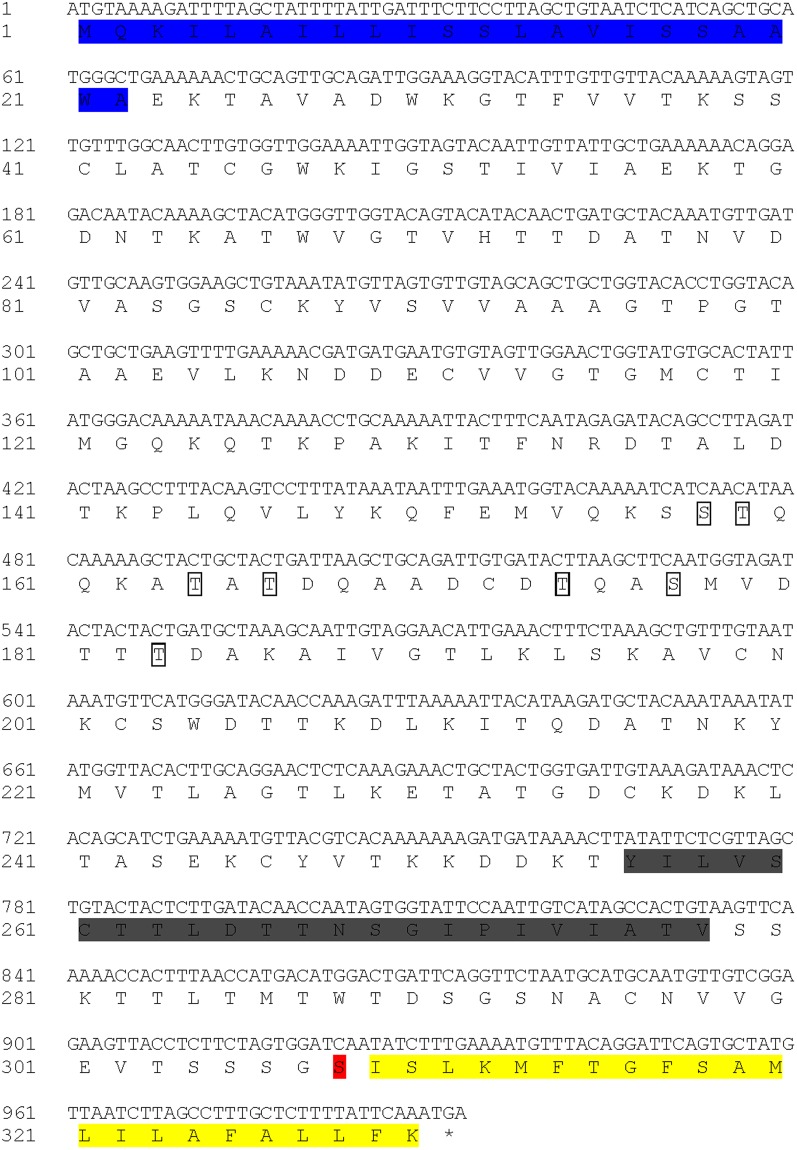
Characteristics of GDCI3 I-antigen gene. The signal peptide, transmembrane region and GPI anchored region were indicated by black letters on a blue, grey, and yellow background, respectively. Seven potential O-glycosylation sites were boxed. The potential ω-site was indicated by red background.

The GDCI3 i-antigen coding sequence was modified so as to target the expressed protein to the culture supernatant by removing the coding sequence for the GPI anchor and adding a sequence for a tandem FLAG/10 × His tag at its new C-terminus (S. Figure 1). The resulting construct was then cloned into a unique NotI site in the ribosomal DNA vector, pD5H8, using previously described methods. Following transformation of *E.coli* (DH5α), plasmid DNA containing the cloned GDCI3 i-antigen insert was isolated and introduced into *Tetrahymena thermophila* as described below.

### Tetrahymena cell transformation

*T. thermophila* strains CU427 (mating type 6) and CU428 (mating type 7) were obtained from the Tetrahymena Stock Center (Cornell University) and cultured separately in NEFF medium (0.25% Protease peptone, 0.25% Yeast extract, 0.55% Dextrose and 33uM FeCl_3_), at 30 °C for 24 hours with shaking (80 rpm). When cells reached a density of ~5 × 10^5^ cells/ml, a total 1.0 × 10^7^ cells of each strain were centrifuged at 800 g for 1.5 minutes. After discarding the medium, cells were suspended in 10 ml of 10 mM Tris-HCl (pH7.4), and incubated at 30 °C for 24 hours with shaking as above. Cells were again counted and adjusted to a final density of 2 × 10^5^ cells/ml. Equal numbers of cells from each culture were then mixed and shaken at 170 rpm at 30 °C for 13 hours, after which the shaker was turned off and the cell incubated an additional Cells were examined microscopically to insure that >80% of cells had undergone mating. During postzygotic mitoses, 1.0 × 10^7^ cells were bombarded with 3 μg plasmid of pD5H8-GDCI3 (coated on 1 mg gold particles) at 900 psi using a Biolistic Particle Delivery System (Bio-Rad, PDS-1000). Following bombardment, cells were cultured in NEFF medium with 10 uM paromomycin and divided into microtiter plates with 100 cells/well. The plates were incubated at 30 °C for 4 days. Wells containing paromomycin-resistant cells were successively transferred into NEFF medium with 20 uM, 50 uM, 100 uM, 150 uM and 200 uM paromomycin to select final transformed cell lines.

### Expression and purification of GDCI3 I-antigen

Positive transgenic *Tetrahymena* cells were cultured in NEFF medium at a density of 5 × 10^5^ cells/ml. After addition of CdCl_2_ to a final concentration of 1 µg/ml, cells were shaken at 30 °C and 5 ml aliquots harvested at 0 h, 1 h, 2 h, 4 h, 6 h, 8 h, 12 h, and 24 h following induction. Expression of recombinant protein at each time point was determined by Western blotting on cell and culture supernatant fractions as described below, using a mouse anti-FLAG monoclonal antibody as the primary screening tool. To obtain the sufficient amounts of GDCI3 i-antigen for further analysis, culture supernatants were harvested 12 hours following induction and a mixture of protease inhibitors (Roche complete, EDTA-free) was added. Recombinant GDCI3 i-antigen containing the 6 × His tag was purified on a nickelnitrilotriacetic acid column (Ni-NTA; Qiagen, Germany), according to the manufacturer’s instructions. To determine the primary structure of GDCI3 i-antigen, the purified band was cut for mass spectrometric analysis.

### Development of rabbit polyclonal antibodies (pAbs) against GDCI3 i-antigen

The purified recombinant GDCI3 i-antigen was emulsified with Freund’s Complete Adjuvant (FCA) (Sigma, USA) and 1 mg protein injected into New Zealand white rabbits (~1.5 kg). Animals were then boosted with 0.5 mg of purified antigen in Freund’s Incomplete Adjuvant (FIA) (Sigma, USA) on two separate occasions. Serum were then prepared and polyclonal antibody (pAb) titers determined by Enzyme-linked immunosorbent assay (ELISA) using recombinant GDCI3 i-antigen. Rabbit IgG were purified from rabbit antiserum using protein G agarose (Beyotime, Haimen, Jiangsu, China) according to the instructions of the manufacturer. Antibody specificity was determined by Western blotting using recombinant GDCI3 i-antigen and total protein from *C. irritans* theronts, trophonts, or tomonts as the antigen.

### Immunofluorescence

For immunofluorescence, *C. irritans* theronts were collected and fixed with immunostaining fixative (Beyotime). After washing twice with PBS, cells were blocked in 1 ml blocking buffer (3% bovine serum albumin in PBS) for 30 minutes at RT. Cells were then concentrated to 0.5 ml and incubated with rabbit anti-GDCI3 antibody (1:2000 dilution) overnight at 4 °C, followed by incubation with fluorescein isothiocyanate (FITC) conjugated goat anti-rabbit IgG (1:500 dilution) for 1 hour at room temperature. DAPI was added at a final concentration of 1 mg/ml for 5 min for nuclear staining. After adding a drop of anti-fade mounting medium to microscope slides (Beyotime), labeled cells were observed and photographed using NIH-Elements System (Nikon, Tokyo, Japan).

### Immunoreactivity of recombinant GDCI3 i-antigen

Purified recombinant GDCI3 i-antigen, rabbit anti-tomont, rabbit anti-theront and rabbit anti-trophont polyclonal antibodies were prepared as previously described^33^. Equal amounts of the purified recombinant GDCI3 i-antigen were fractionated by SDS-PAGE, transferred to filters and detected by Western blotting using rabbit anti-parasite polyclonal antibodies as below described,.

### Grouper immunization and challenge

Orange-spotted groupers (32.8 ± 4.8 g), purchased from the Marine Fisheries Development Center of Guangdong Province, Guangdong, China, were maintained at 27 °C in a flow-through water system (300 L). Groupers were acclimated for two weeks and fed daily with commercial grouper feed. Thirty groupers were injected intraperitoneally (IP) with the purified recombinant GDCI3 i-antigen emulsified with FCA at a protein concentration of 0.3 mg/fish. Fourteen days after the first immunization, groupers were booster-immunized with 0.15 mg/fish GDCI3 i-antigen emulsified with FIA. The control group was injected intraperitoneally with phosphate buffer saline (PBS) emulsified in FCA and FIA in primary and booster immunizations, respectively. Fish were challenged with a dose of 65,000 theronts per fish 28 days after the first immunization. The number of dead fish was recorded every day. The relative percent survival (RPS) was calculated according to the following formulation, RPS (%) = [1 − (mortality rate in the immunized group/mortality rate in the control group)] × 100%. Serum and mucus from the immune and control groupers were collected at day 28 (before challenge) and day 30 (two days after challenge).

### Immobilization assay

Serum from the immune and control groupers collected at day 30, and rabbit anti-GDCI3 i-antigen IgG as well as normal rabbit IgG (dissolved in sterilized sea water) were used to perform the immobilization assays according to the method described by Luo *et al*.^5^ with minor modifications. Briefly, serum was incubated at 56 °C for 30 min for inactivation of complement, 50 ul fish serum or rabbit antibody was subjected to serial two-fold dilutions with sterilized sea water in a 96-well plate. Fifty ul sea water contain 500 theronts was then added to each well and incubated for 30 min at RT. Immobilization titers were determined as the last well in which 50% of the cells were immobilized.

### Non-specific and parasite-specific IgM detection

Levels of non-specific antibodies were determined by subjecting equal loadings of diluted serum (1:10 dilution) or mucus (1:2 dilution) from immunized and control groupers to SDS-PAGE and Western blotting with anti-grouper monoclonal antibody as described below. Levels of specific antibodies were determined following binding of sera to *C. irritans* theronts as described by Xu *et al*.^34^ with minor modifications. Briefly, theronts were harvested and 50 ul of cells (~20,000 theronts) were incubated with either 1:10 or 1:2 dilutions of serum or mucus, respectively, from immune and control groupers at 4 °C for 2 hours with shaking. Theronts were then washed with PBS five times. Theronts with bound antibody were then boiled in SDS sample buffer and analyzed by Western blotting with anti-grouper monoclonal antibody. Signals from immunoblots were quantitated by densitometry using Image J software, and presented as relative to values of control fish (n = 3).

### Western blotting

Equal amounts of various protein samples were electrophoresed on 10% SDS-PAGE gels and subsequently transferred to polyvinylidene fluoride (PVDF) membranes. The PVDF membranes were blocked in 10% dried milk (dilute in PBST) for 1 hour, followed by incubation with rabbit anti-GDCI3 (1:2000 dilution), rabbit anti-trophont (1:500 dilution), rabbit anti-tomont (1:500 dilution), rabbit anti-theront (1:500 dilution) polyclonal antibody or mouse anti-grouper IgM monoclonal antibody (1:2000 dilution) overnight at 4 °C, respectively. Membranes were washed with PBST three times and incubated with secondary antibodies (either goat anti-rabbit IgG antibodies conjugated to horse radish peroxidase (HPR), or goat anti-mouse IgG conjugated to HPR) for 1 hr at room RT, respectively. Membranes were washed in PBST three times and incubated with by SuperSignal West Pico Chemiluminescent Substrate (Thermo), then exposed and analyzed using Tanon 5200 chemiluminescence imaging analysis system (Tanon).

## Results

### Characteristics and analysis of GDCI3 I-antigen gene

Analysis of transcriptome data from all stages of *C. irritans* revealed nine putative i-antigen transcripts with one, encoding the GDCI3 i-antigen, showing the highest level of expression. As determined following 5′-RACE, the complete open reading frame of GDCI3 i-antigen cDNA (GenBank no. MF521599) was 993 bp, specifying 330 deduced amino acids with a theoretical molecular mass of 34.8 kDa (Fig. 1). The GDCI3 i-antigen contains a predicted signal peptide at its N-terminus, a possible transmembrane domain, as well as a predicted GPI anchor at its C-terminus. Two potential ω-(cleavage) sites were predicted (S304 and S308), with S308 having a higher score. There were no predicted N- or O-glycosyl modifications in the protein. The amino acid sequence of GDCI3 i-antigen was 98% identical to that of the previously annotated *C. irritans* agglutination/immobilization antigen precursor (GenBank no. ACN89783) which isolated from marine fishes in Taiwan (Unpublished).

### Expression analysis GDCI3 I-antigen in *C. irritans*

**Figure 2:**
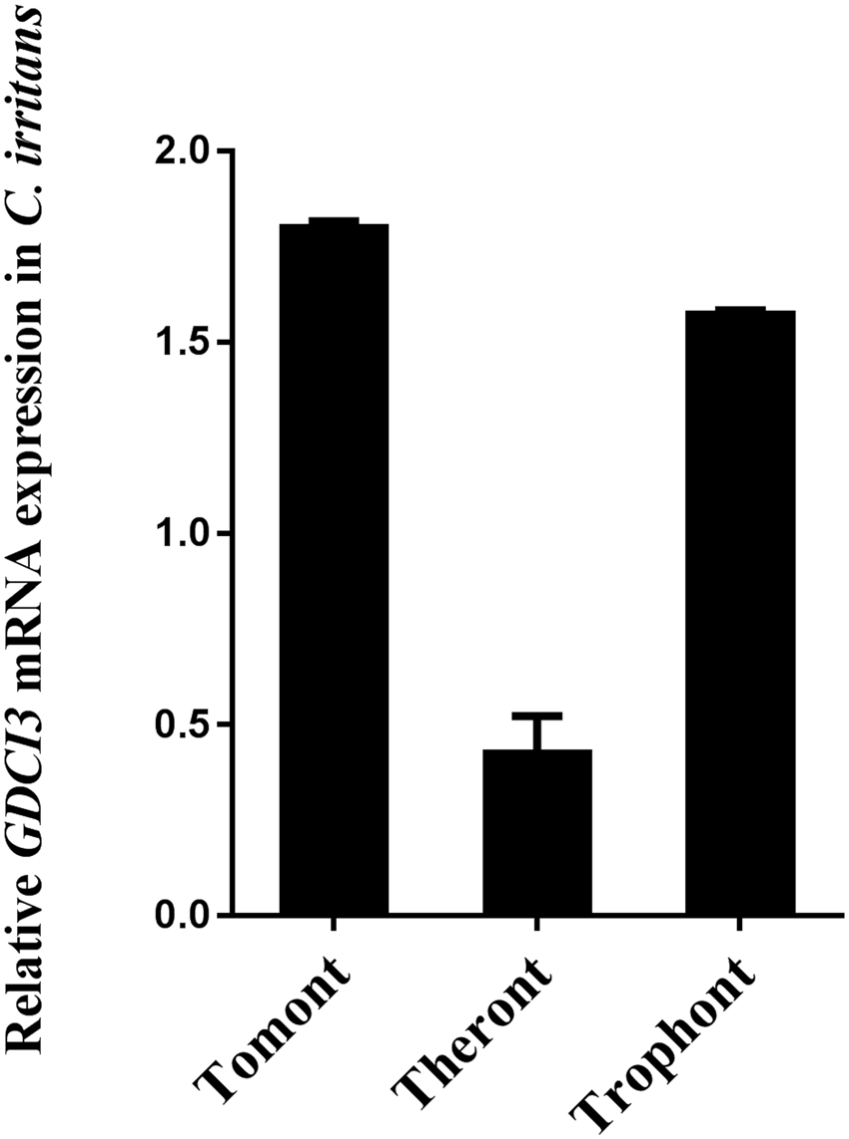
Expression analysis of GDCI3 I-antigen gene in *C. irritans*. *C. irritans* EF-1 β was introduced as the reference gene. The GDCI3 I-antigen’s mRNA is expressed in all three stages of *C. irritans* and highest expressed in trophont.

GDCI3 i-antigen transcripts were detected in all three stages of *C. irritans* by RT-PCR (Fig. 2), with the highest level occurring in trophonts followed by tomonts and theronts. These data mimic the relative expression patterns of GDCI3 mRNAs predicted from transcriptomics data reported previously^31^. Western blotting using rabbit antiserum against the purified recombinant antigen also demonstrated that the corresponding parasite protein is expressed in all three stages (see below).

### Production of recombinant GDCI3 I-antigen in *Tetrahymena*

**Figure 3:**
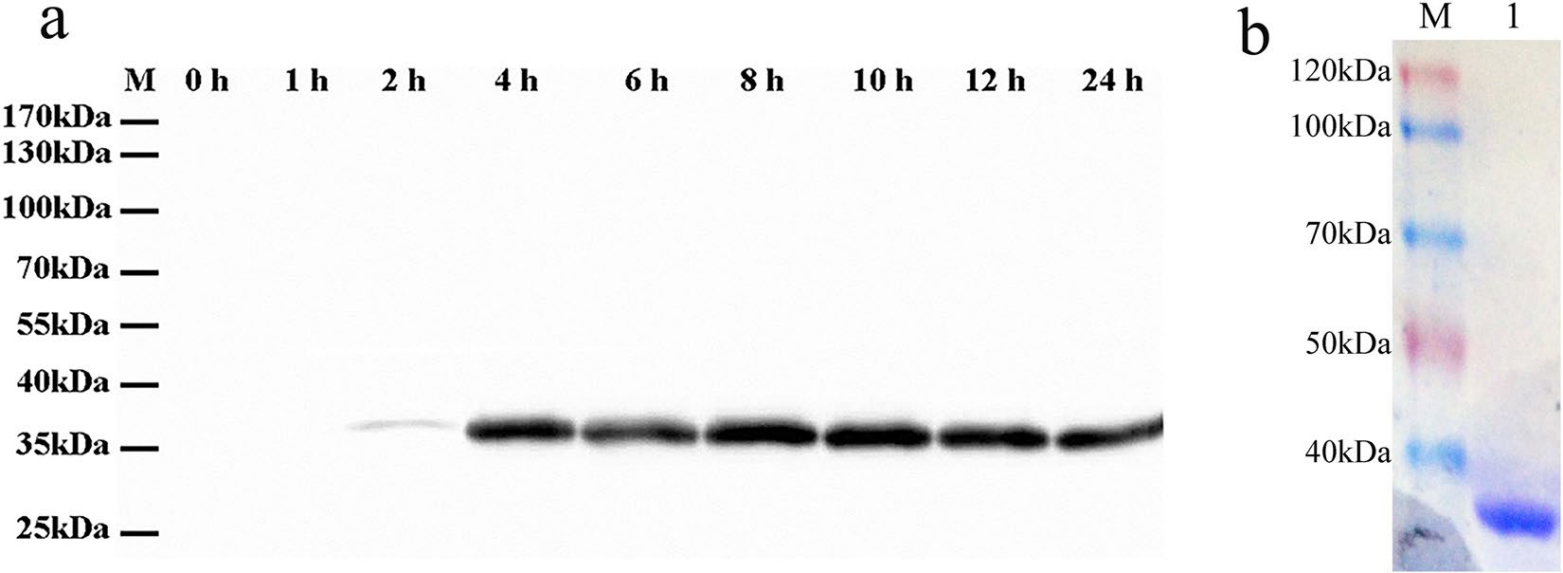
Expression and purification of GDCI3 I-antigen. (**a**) Western blotting of cultural supernatant harvested at 0 h, 1 h, 2 h, 4 h, 6 h, 8 h, 12 h, and 24 h post the treatment of CdCl_2_. A mouse anti-FLAG monoclonal antibody was used as primary antibody, HRP-conjugated goat anti-mouse as second antibody. (**b**) SDS-PAGE analysis of the purified recombinant GDCI3 I-antigen. An ~36 kDa target band was observed. Line 1: the purified recombinant GDCI3 I-antigen protein. M: Protein marker.

Analysis of codon usage preferences in *C. irritans* and *T. thermophila* suggested that *Tetrahymena* would be a suitable host for expression of *Cryptocaryon* genes (S. Figure 2). To express the GDCI3 i-antigen in *T. thermophila*, a cDNA construct encoding a tagged version of the full-length antigen minus the GPI anchor was cloned into a high copy ribosomal DNA vector under the control of a cadmium inducible promoter and introduced into *Tetrahymena* via biolistic bombardment^35^. In the absence of the C-terminal glycolipid anchor the recombinant protein was expected to traffic to the extracellular space^36^. As shown in Fig. 3a, beginning 2 h following induction, a single band migrating at ~36 kDa could easily be detected by Western blotting in culture supernatant fractions derived from transformed cells. The protein was subsequently purified by affinity chromatography (Fig. 3b) and used for further study. As shown by mass spectrometry, 12 peptides from the purified protein matched sequences in the predicted GDCI3 i-antigen, scoring 249, and comprising 38% of the sequence (S. Figure 3). These results confirmed that the protein purified from the culture supernatant was, in fact, the GDCI3 i-antigen.

### Development of anti-GDCI3 antibodies and subcellular localization of GDCI3 I-antigen in *C. irritans*

**Figure 4:**
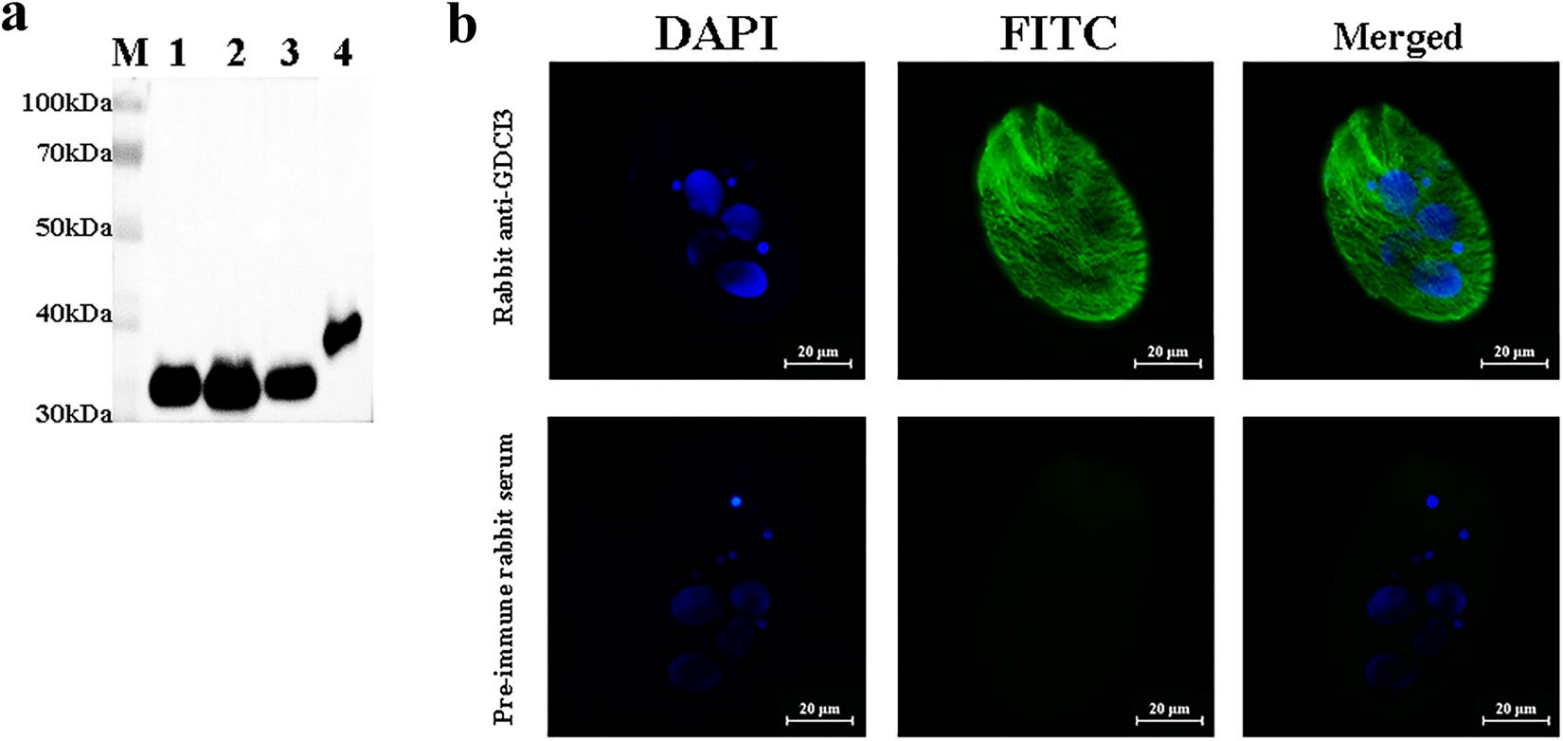
The specificity of rabbit anti-GDCI3 I-antigen antibody and the subcellular localization of GDCI3 I-antigen in *C. irritans*. (**a**) Western blotting using rabbit anti-GDCI3 I-antigen antibody as primary antibody, HRP-conjugated goat anti-rabbit as second antibody. Total protein from tomont, theront, trophont, and the purified recombinant GDCI3 I-antigen protein were used as antigen, respectively. M: Protein marker. (**b**) Immunofluorescence of theronts stain with rabbit anti-GDCI3 I-antigen antibody. Pre-immune rabbit serum was used as control. Scale bar was 20 um.

**Figure 5:**
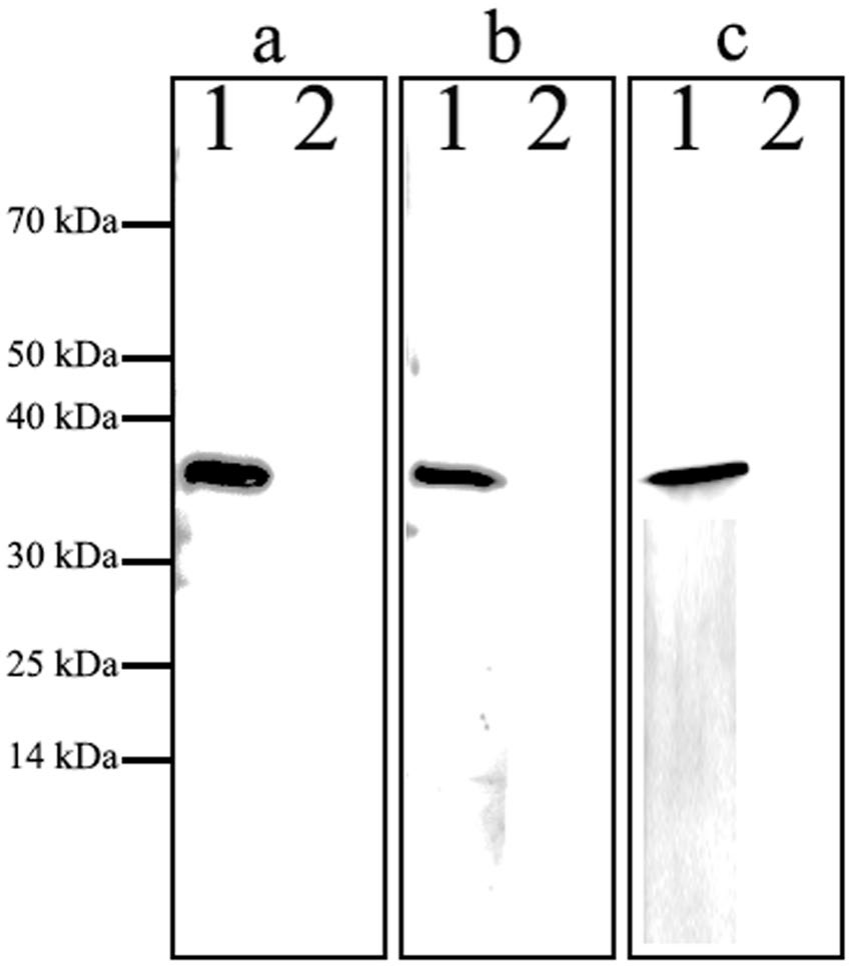
Immunoreactivity analysis of GDCI3 I-antigen. Western blotting using rabbit anti-tomont (**a**), rabbit anti-theront (**b**) and rabbit anti-trophont (**c**) polyclonal antibody as primary antibody, HRP-conjugated goat anti-rabbit as second antibody. Line 1: The purified recombinant GDCI3 I-antigen protein. Line 2: Culture supernatant of transgenic *Tetrahymena* cells without CdCl_2_ treatment. M: Protein marker.

High-titer antiserum were generated in rabbits by injecting animals with the purified GDCI3 antigen. The specificity of resulting antibodies was confirmed in Western blots showing only a single band of ~32 kDa when total protein from *C. irritans* tomonts, theronts and trophonts were screened (Fig. 4a). Immunofluorescence analysis of fixed cells showed that GDCI3 i-antigen was diffusely localized in *C. irritans* (Fig. 4b) with a preponderance of staining at the cell periphery. As expected, polyclonal rabbit IgG against *C. irritans* trophonts, tomonts, and theronts, respectively, all recognized the recombinant GDCI3 i-antigen in Western blots (Fig. 5).

### Immunization of fish

**Figure 6:**
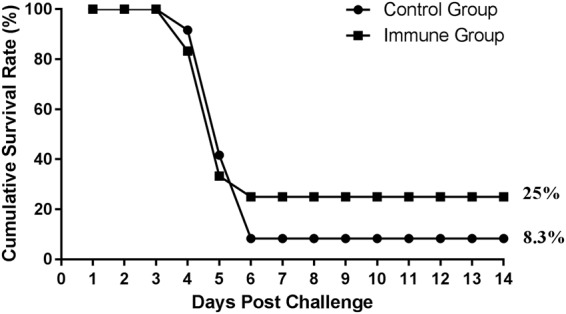
Cumulative survival rate post the challenge. The immune and control groupers were challenged with a dose of 65,000 theronts per fish 28 days after the first immunization. Death fish number was record daily and presented as cumulative survival rate (%). The cumulative survival rate of the immune group was 25% while the control group was 8.3%.

To determine whether the recombinant GDCI3 i-antigen could induce a protective immune response in fish, orange-spotted groupers were vaccinated with recombinant protein and both immune and control groups challenged with a lethal dose of parasites 28 days after the first immunization. Following challenge, fish in all groups showed typical symptoms of small white spots on their skin, fins, and gills. Deaths began to occur on day 4 after challenge and plateaued on day 6. On day 14 (when the experiment was terminated), groupers immunized with recombinant GDCI3 i-antigen showed an 18% relative percent survival compared to controls injected with PBS alone (Fig. 6).

### Immobilization assays

**Figure 7:**
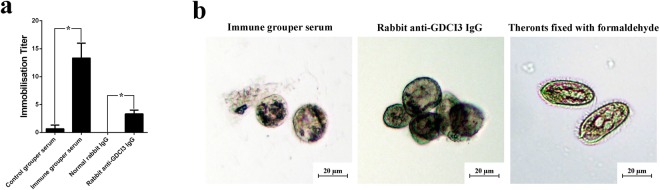
Immobilization assay. (**a**) Immobilization titer of groupers serum and rabbit IgG (n = 3). Significant increase immobilization titer is indicated with *(p < 0.05). (**b**) Immobilized theronts incubated with immune groupers serum (left) or rabbit anti-GDCI3 I-antigen IgG (middle), respectively. Theronts fixed with formaldehyde were used as control (right).

To determine whether antibodies generated in animals were capable of immobilizing *C. irritans*, live theronts were incubated in sera from either groupers or rabbits immunized with the recombinant GCDI3 i-antigen. Clusters of immobilized theronts were clearly visible within 30 min of incubation in low dilutions of either immune grouper serum or rabbit anti-GDCI3 i-antigen IgG. As shown in Fig. 7a, immobilization titers were determined to be 13.3 ± 4.6 and 3.3 ± 1.1, for fish serum and rabbit IgG, respectively. Additionally immobilized and aggregated theronts showed a rounded morphology (Fig. 7b). By contrast theronts incubated in control grouper serum or non-immune rabbit IgG appeared normal in all respects.

### Serum and mucus antibodies responses in immunized fish

**Figure 8:**
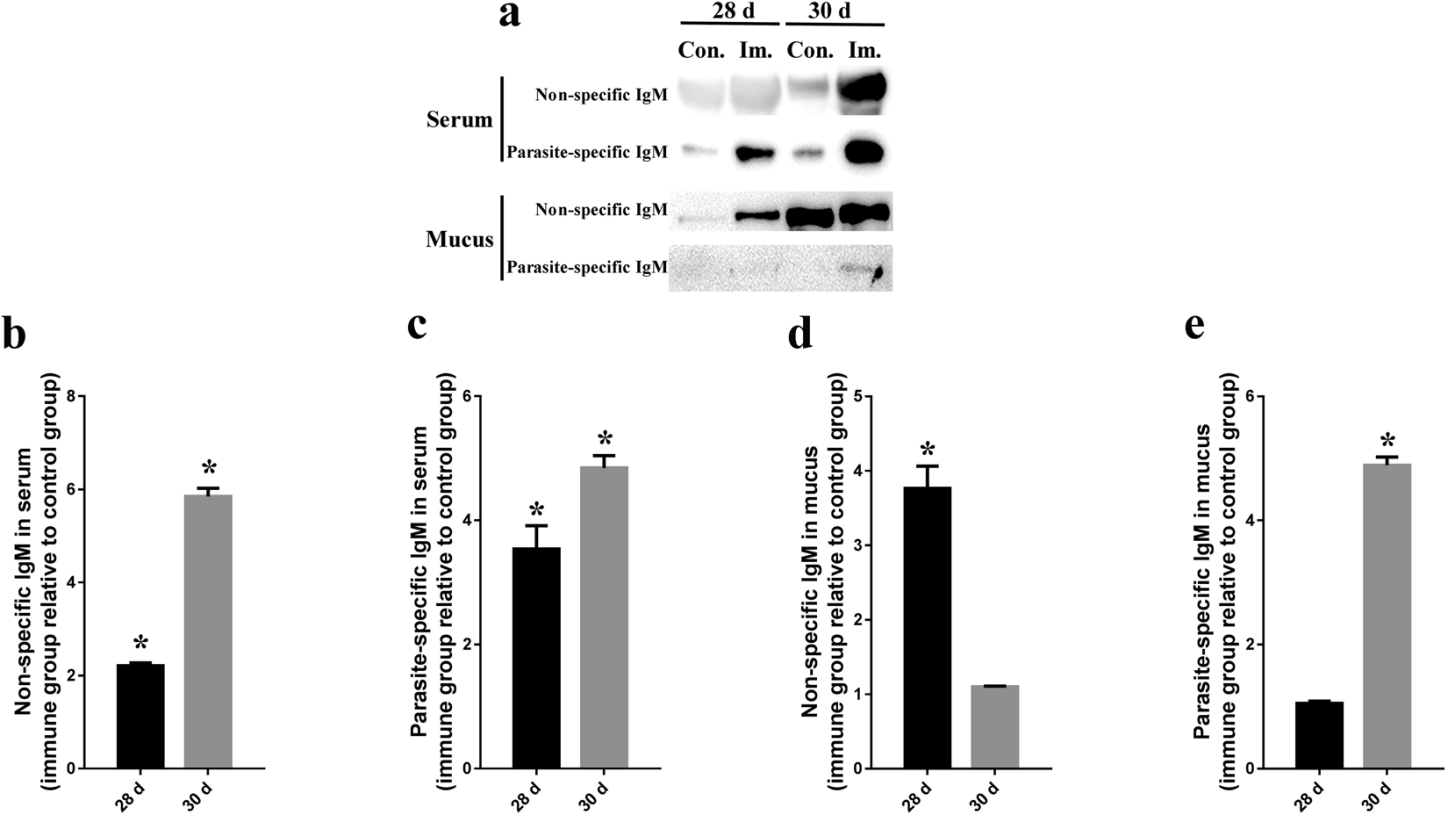
Antibody raising in immune grouper serum and mucus. Serum and mucus from the immune and control groupers were collected at day 28 (before challenge) and day 30 (two day after challenge). (**a**) The non-specific and parasite-specific IgM against *C. irritans* were detected using Western blotting with anti-grouper IgM antibody. Con.: Control group; Imm.: Immune group. (**b**–**e**) The non-specific and specific IgM were measured by densitometric analysis of immunoblots and presented relative to values of control fish (n = 3). Significant increase between immune and control group is indicated with *(p < 0.05).

To study parasite-specific and non-specifc IgM responses in groupers following vaccination and parasite challenge, serum and mucus were collected from controls (sham vaccinated) and animals vaccinated with GDCI3 i-antigen 28 days after immunization, and again two days following infection with live theronts (day 30). As expected, parasite specific antibodies showed a marked increase in serum following vaccination and challenge (Fig. 8a,c). Somewhat surprisingly, increases in non-specific IgMs were also seen (Fig. 8a,b). In the case of mucus, parasite-specific increases in IgM were seen following challenge, but were less clear at day 28 in the immunized fish (Fig. 8a,e). Again somewhat surprisingly, non-specific IgMs appeared elevated in mucus of vaccinated animals on day 28 (Fig. 8a,d), and were further elevated after challenge.

## Discussion

Immobilization antigens have long been considered promising vaccine candidates against *C. irritans*^24,25^. Previously, we sequenced the transcriptome of *C. irritans* and nine immobilization antigen ESTs were identified^31^. The most highly expressed i-antigen transcript was then cloned and sequenced. As shown here, its deduced amino acid sequence is roughly 98% identical to that of a *C. irritans* agglutination/immobilization antigen precursor described elsewhere (GenBank no. ACN89783) and predicts an N-terminal signal peptide and C-terminal signal sequence for addition of a GPI anchor, which are typical of ciliate immobilization antigens more generally^17,18,21,24,25^. Unlike the i-antigens of other ciliates, GDCI3 i-antigen contains a predicted transmembrane domain in addition to its GPI-anchor, although it is unclear whether this domain actually spans the membrane given that the recombinant protein lacking the C-terminal signal sequence was secreted in *T. thermophila*. Based on its mRNA expression patterns the GDCI3 i-antigen appears to be made in all three stages of the *C. irritans* life cycle (Fig. 2). This was confirmed at the protein level by Western blotting using rabbit anti-GDCI3 antibody (Fig. 4). Furthermore, as expected, the GDCI3 protein appears to be localized predominantly at the surface of *C. irritans* theronts (Fig. 4b). Taken together, we speculated that the GDCI3 i-antigen was suitable for vaccine development. Notably, it is 98% (other than 100%) identical between GDCI3 and the i-antigen isolated from Taiwan (ACN89783), while both sequences own the same feature. Recent study on the phylogenetic analyses of *C. irritans* from 8 new isolates of *C. irritans* sampled showed that *C. irritans* consists of at least two genetically distinct groups/populations^37^. Given that the GDCI3 was isolated in Guangdong, while ACN89783 was isolated in Taiwan, it’s more likely that these two sequences belong to the same gene, but come from two different isolates of *C. irritans*. Due to lack of other *C. irritans* isolates, whether GDCI3 or other i-antigens can be used as a vaccine against *C. irritans* isolation from different areas still needs further study.

*Tetrahymena* is an important model for cellular biology and an excellent expression system for heterologous proteins^36,38,39^ with numerous advantages for the production of subunit vaccines. Hence, in this study, the *Tetrahymena* expression system was used to produce the recombinant GDCI3 i-antigen protein. To simplify purification procedures and increase the yield of recombinant protein, we modified the GDCI3 i-antigen cDNA by deleting the coding region for the GPI fragment at its C-terminus with the expectation that the resulting protein product would be secreted from cells. Indeed, a secreted version of GDCI3 was successfully expressed and the purified recombinant antigen recognized by all rabbit anti-*C. irritans* antibodies demonstrating cross-reactivity of the truncated protein with the native parasite i-antigen. On a somewhat cautionary note, however, immobilization assays with serum or purified IgGs from animals immunized with the recombinant secreted protein were only weakly positive (Fig. 7) compared with serum from fish immunized with whole theronts^5^.

Despite the relatively low immobilization titers, use of the recombinant protein for vaccination studies were prompted by a recent report showing that a codon optimized i-antigen DNA vaccine boosted with a bacterially expressed recombinant i-antigen protein provided 40% relative percent survival in groupers challenged with a lethal dose of *C. irritans* theronts^27^. Remarkably, a follow-on study using heat shock protein 70 cloned from *C. irritans* as an adjuvant along with an i-antigen DNA vaccine administered orally to intubated grouper fingerlings showed a 100% relative percent survival after challenge^28^. This is considerably higher than the 18% relative percent survival we found using the purified recombinant antigen administered IP. The lower level of protection reported here with the recombinant secreted antigen is somewhat consistent with the low immobilization titers in serum from immunized fish. Nevertheless, in view of the crucial role of adjuvants and the route of delivery in generating immune protection, it is possible that improved adjuvants or oral intubation would boost the activity of the recombinant protein. Alternatively, the truncated antigen used here lacks the GPI-anchor (which itself may serve as a powerful adjuvant^40^), or may lack critical epitopes present in the native protein responsible for protection. Finally, regardless of the findings with i-antigen-based DNA vaccines^27,28^, additional i-antigens, or entirely different proteins for that matter, may be responsible for the protection generated by whole live parasites.

Interestingly, both non-specific and parasite-specific IgMs were elicited by immunization of groupers with the recombinant GDCI3 i-antigen, and in both cases those IgMs were elevated following challenge (day 30). The increase in non-specific serum IgM in infected fish is likely to reflect broad activation of B-cell responses although it is not clear why this is so much more pronounced in the immunized fish. Similar increases in non-specific IgM in mucus were also seen, However, parasite-specific IgM in mucus could only be detected after challenge of immunized fish. Since IgM is unlikely to transfer from serum to mucus within two days of infection, we speculate that intraperitoneal injection of recombinant GDCI3 i-antigen may allow groupers to generate memory B cells in skin that secrete antibodies to the mucus when infection ensues. Regardless, parasite-specific IgMs were difficult to detect even in the immunized fish, which may also account for the low protection seen here.

## Electronic supplementary material

Supplementary
