# Supplementary material for: Characterization and immune regulation role of an immobilization antigen from Cryptocaryon irritans on groupers
Source: Sci Rep. 2019 Jan 31;9:1029. doi: 10.1038/s41598-018-25710-3 (PMC6355922; doi:10.1038/s41598-018-25710-3)

**Characterization and immune regulation role of an immobilization antigen from *Cryptocaryon irritans* on groupers**

Ze-Quan Mo ^a^, Shun Xu ^b^, Donna M. Cassidy-Hanley ^c^, Yan-Wei Li ^a^, Daniel Kolbin ^c^, Jennifer M. Fricke ^c^, An-Xing Li ^d^, Theodore G. Clark ^c^*, Xue-Ming Dan ^a^**

*^a^Joint Laboratory of Guangdong Province and Hong Kong Regions on Marine Bioresource Conservation and Exploitation, College of Marine Sciences, South China Agricultural University, Guangzhou, 510642, China*

*^b^School of Bioscience and Bioengineering, South China University of Technology, Guangzhou 510006, PR China*

*^c^Department of Microbiology and Immunology, College of Veterinary Medicine, Cornell University, Ithaca, NY 14853, USA*

*^d^State Key Laboratory of Biocontrol/ Key Laboratory of Aquatic Product Safety (Sun Yat-Sen University), Ministry of Education, The School of Life Sciences, Sun Yat-sen University, Guangzhou 510275, Guangdong Province,* *PR China*

Ze-Quan Mo: [465260412@qq.com](mailto:465260412@qq.com); Shun Xu: [1548045443@qq.com](mailto:1548045443@qq.com); Donna M. Cassidy-Hanley: dmc4@cornell.edu; Yan-Wei Li: [yanweili@scau.edu.cn](mailto:yanweili@scau.edu.cn); Daniel Kolbin: dk99@cornell.edu; Jennifer M. Fricke: jmf368@cornell.edu; An-Xing Li: [lianxing@mail.sysu.edu.cn](mailto:lianxing@mail.sysu.edu.cn)

** Correspondence to: X.-M. Dan, College of Marine Sciences, South China Agricultural University, 483 Wushan Street, Tianhe District, Guangzhou 510642, PR China. Tel. /fax: +86 20 85283529. E-mail: [dxm72@scau.edu.cn](mailto:dxm72@scau.edu.cn)

* Correspondence to: T.G. Clark, Department of Microbiology and Immunology, College of Veterinary Medicine, Cornell University, Ithaca, NY 14853, USA Tel.: +1 607 253 4042; fax: +1 607 253 3384. E-mail: [tgc3@cornell.edu](mailto:tgc3@cornell.edu)

**Table 1 The expression level of immobilization antigen in *Cryptocaryon irritans*.**

|  | Gene annotation using NR database | Tomont (RPKM) | Theront (RPKM) | Trophont (RPKM) |
| --- | --- | --- | --- | --- |
| immobilization antigen GDCI 1 | gi\|325656982\|gb\|ADZ38984.1\|:"immobilization antigen, partial [Cryptocaryon irritans]" | 0.05 | 2.82 | 0.25 |
| immobilization antigen GDCI 2 | gi\|332273842\|gb\|AEE39297.1\|:agglutination/immobilization antigen [Cryptocaryon irritans] | 488.54 | 8.84 | 1667.82 |
| immobilization antigen GDCI 3 | gi\|225420798\|gb\|ACN89783.1\|:agglutination/immobilization antigen precursor [Cryptocaryon irritans] | 1773.37 | 394.64 | 3819.62 |
| immobilization antigen GDCI 4 | gi\|325656984\|gb\|ADZ38985.1\|:"immobilization antigen, partial [Cryptocaryon irritans]" | 0.08 | 5.43 | 1.03 |
| immobilization antigen GDCI 5 | gi\|171854877\|dbj\|BAG16623.1\|:agglutination/immobilization antigen [Cryptocaryon irritans] | 1019.34 | 218.39 | 3075.68 |
| immobilization antigen GDCI 6 | gi\|482679741\|gb\|AGK30041.1\|:agglutination/immobilization antigen [Cryptocaryon irritans] | 103.14 | 10.11 | 3491.39 |
| immobilization antigen GDCI 7 | gi\|330369972\|gb\|AEC12209.1\|:agglutination/immobilization antigen [Cryptocaryon irritans] | 45.68 | 9.09 | 4.55 |
| immobilization antigen GDCI 8 | gi\|118596620\|dbj\|BAF37970.1\|:agglutination/immobilization antigen isoform 2 [Cryptocaryon irritans] | 0.34 | 359.20 | 39.13 |
| immobilization antigen GDCI 9 | gi\|225420796\|gb\|ACN89782.1\|:agglutination/immobilization antigen precursor [Cryptocaryon irritans] | 1.95 | 44.28 | 54.15 |

**Figure. 1 Illustrator of** **pTIEV4-GDCI3 shuttle vector.**
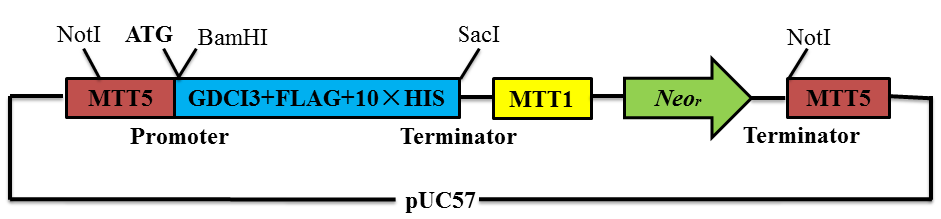


**Figure 2 The differences of GDCI3 I-antigen codon usage preference between *C. irritans* and Tetrahymena were analyzed with GCUA tool.**

**
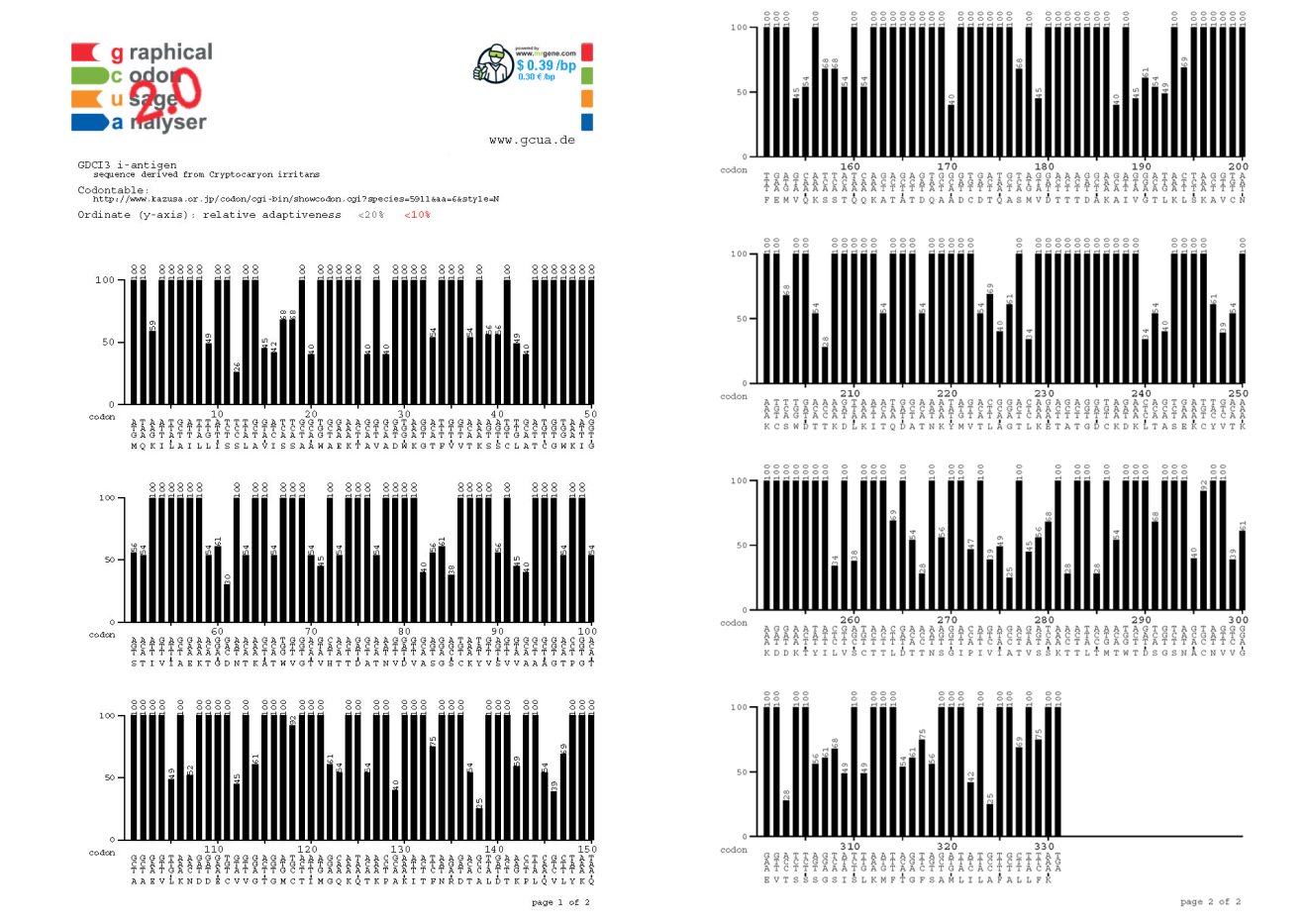
**

**Figure 3 Mass spectrometric analysis of** **purified GDCI3 I-antigen.**

**
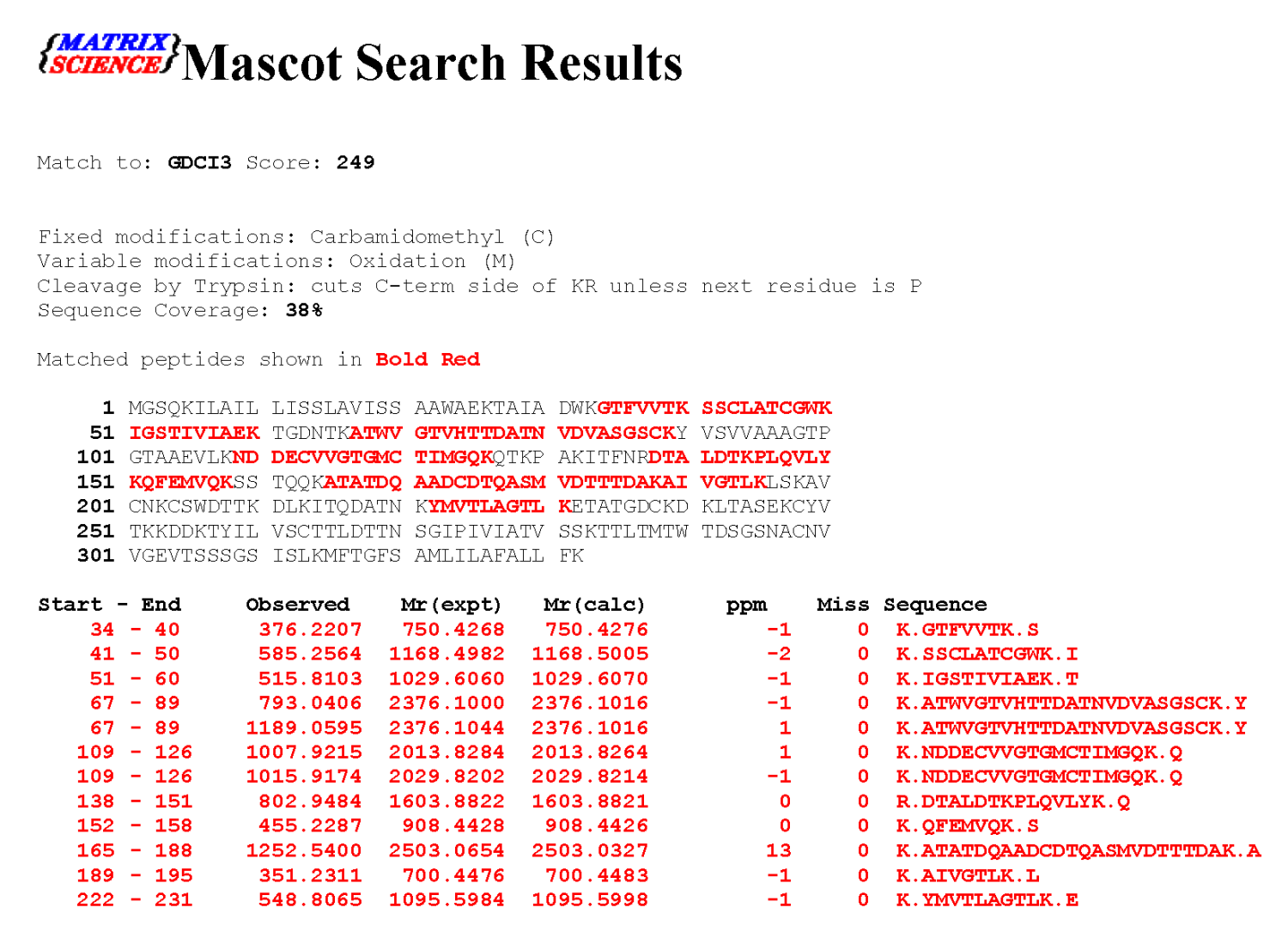
**

**Figure 8A-1 full-length blot**


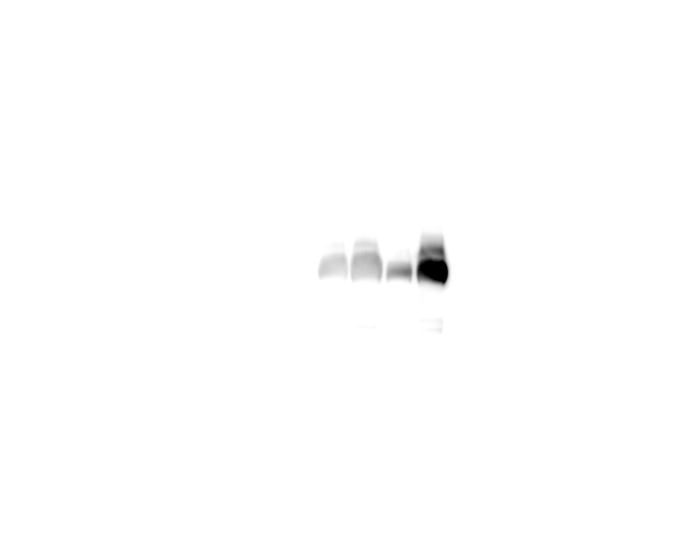


**Figure 8A-2 full-length blot**


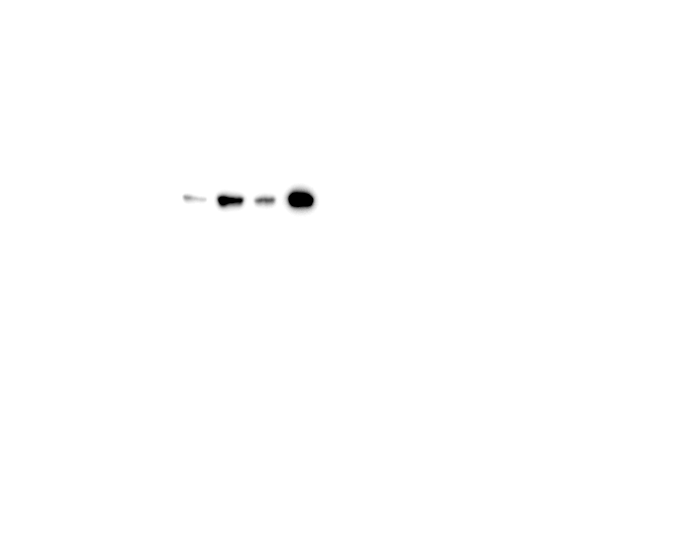


**Figure 8A-3 full-length blot**


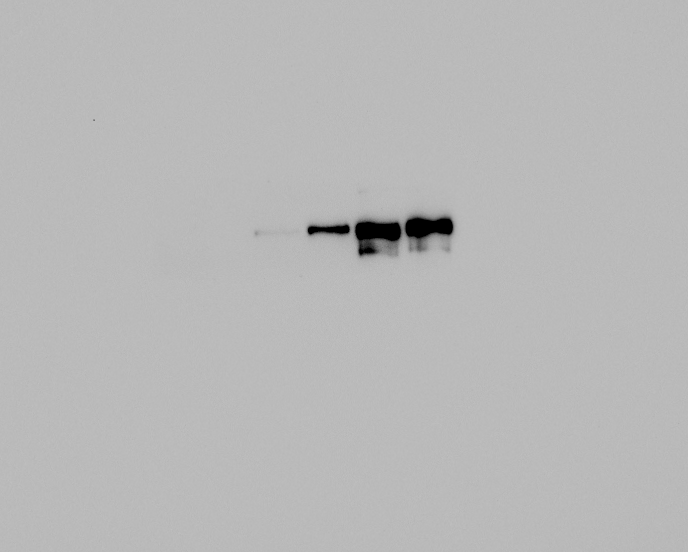


**Figure 8A-4 full-length blot**


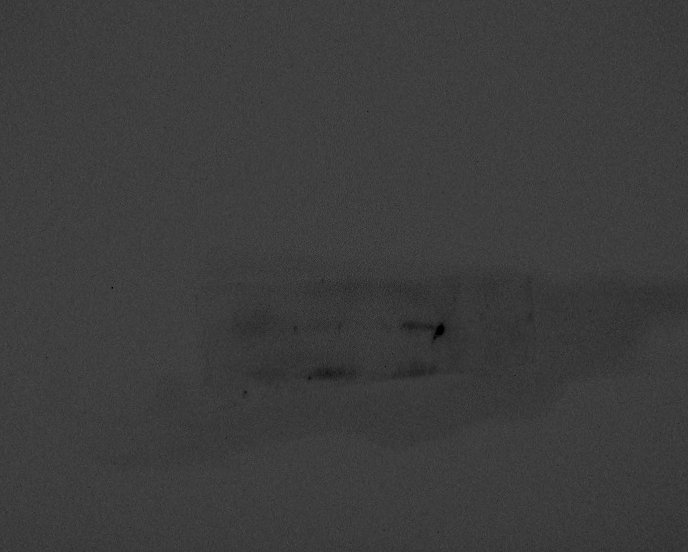

Supplement: Supplementary file 1 — Supplementary [file 41598_2018_25710_MOESM1_ESM.docx]
